# Altered Brain Signal Variability in Patients With Generalized Anxiety Disorder

**DOI:** 10.3389/fpsyt.2019.00084

**Published:** 2019-03-04

**Authors:** Liyuan Li, YiFeng Wang, Liangkai Ye, Wang Chen, Xinju Huang, Qian Cui, Zongling He, Dongfeng Liu, Huafu Chen

**Affiliations:** ^1^The Clinical Hospital of Chengdu Brain Science Institute, MOE Key Lab for Neuroinformation, University of Electronic Science and Technology of China, Chengdu, China; ^2^School of Life Science and Technology, Center for Information in BioMedicine, University of Electronic Science and Technology of China, Chengdu, China; ^3^School of Public Affairs and Administration, University of Electronic Science and Technology of China, Chengdu, China; ^4^Mental Health Center, The Fourth People's Hospital of Chengdu, Sichuan, China

**Keywords:** brain signal variability, fMRI, generalized anxiety disorder, neurodynamics, non-linear relationship

## Abstract

Generalized anxiety disorder (GAD) is characterized by a chronic, continuous symptom of worry and exaggerated startle response. Although functional abnormality in GAD has been widely studied using functional magnetic resonance imaging (fMRI), the dynamic signatures of GAD are not fully understood. As a vital index of brain function, brain signal variability (BSV) reflects the capacity of state transition of neural activities. In this study, we recruited 47 patients with GAD and 38 healthy controls (HCs) to investigate whether or not BSV is altered in patients with GAD by measuring the standard deviation of fMRI signal of each voxel. We found that patients with GAD exhibited decreased BSV in widespread regions including the visual network, sensorimotor network, frontoparietal network, limbic system, and thalamus, indicating an inflexible brain state transfer pattern in these systems. Furthermore, the correlation between BSV and trait anxiety score was prone to be positive in patients with GAD but negative in HCs. The opposite relationships between BSV and anxiety level in the two groups indicate that the brain with moderate anxiety level may stay in the most stable rather than in the flexible state. As the first study of BSV in GAD, we revealed extensively decreased BSV in patients with GAD similar to that in other mental disorders but with a non-linear relationship between BSV and anxiety level indicating a novel neurodynamic mechanism of the anxious brain.

## Introduction

Generalized anxiety disorder (GAD) is one of the most prevalent mental disorders characterized by exaggerated startle response and chronic, pervasive, and intrusive worry (1, 2). Based on static analysis methods, many functional magnetic resonance imaging (fMRI) studies have found aberrant brain activation related to cognition and emotion functions in GAD (3, 4). Inefficient intrinsic brain activity associated with integration of interoceptive and somesthetic functions has also been found in anxiety disorders (5–7). These static analysis methods for brain activity or activation have provided abundant evidence for us to understand the neural mechanism of GAD. However, clinically effective biomarker is still lacking. Recently, a mass of studies have shown that dynamic brain activity can provide novel information of neural characteristics for various neural disorders (8–10). Whether the dynamic brain activity analysis can provide insightful information about the neural mechanism of GAD, however, is unknown.

Human brain activity is naturally variable (11). In previous years, fMRI research had regressed blood oxygen level-dependent (BOLD) signal variance as measurement-related or other confounds (12, 13). However, researchers found that the “noise” variance in data is an important feature of brain function in the recent 10 years (14, 15). In a neuroimaging time series, BSV measures the magnitude of variability from moment to moment (16). The forms of BSV include variance (17) and mean square successive differences (18), especially standard deviation (SD) (14, 15). As the next frontier in brain mapping, the brain signal variability (BSV) reflects the capacity of state transition of neural activities and dynamic range of brain functional systems (16). BSV has been suggested to be an excellent proxy of the characteristics of neural dynamics, cognitive performance, and brain disorders (14, 19–21). Great BSV has been suggested to be associated with increased ability to transfer between brain states (22) and to process varying and unexpected external stimuli (16, 23). Measured with the SD of brain signal, the BSV is more powerful than mean brain signal in predicting neural aging (14). Recently, the quadratic change in lifespan BSV trajectory has been further uncovered (24). Furthermore, a number of studies have demonstrated abnormal BSV in schizophrenia, attention deficit hyperactivity disorder, autism, and patients with disorders of consciousness, reflecting the dynamical dysfunction of neural activities in mental disorders (25, 26). Specifically, the non-linear dynamics of brain signal over a range of temporal scales are mainly decreased compared with those in healthy controls (HCs) (27). The hypothesis of “unhealthy brain is less variable than healthy brain” has been demonstrated in various clinical populations, such as dementia, untreated patients with schizophrenia, autism, and mesial temporal lobe epilepsy (16, 27, 28). In other words, many findings support Pool's opinion that “healthy brain is a chaotic brain” (11).

The anxious brain was viewed as an inflexible system, grounded in poor inhibition (29). In patients with GAD, a reduced capacity to inhibit cognitive (worry), behavioral (avoidance), and accompanying physiological manifestations was associated with cognitive rigidity and inflexibility (30). By using the mean-based methods, the core symptom, worry, which predominantly reflected a stimulus-independent mental processing also leads to the inflexible functional brain configurations in the prefrontal cortex, cingulate gyrus, and amygdala (31). However, the respective spatial patterns profiled by the SD-based method (like BSV) and mean-based method were highly different (14). To profile the flexibility of brain, BSV is a brand new effective index. Therefore, we hypothesized that patients with GAD may show decreased BSV compared with HCs in more related core regions.

In this study, we investigated the altered BSV in patients with GAD and healthy participants. To perform comprehensive comparisons, the relationship between BSV and anxiety level was also observed. Notably, this work is the first study of altered BSV in patients with GAD. Therefore, the BSV may be able to provide new insights into understanding the neural dynamics of GAD.

## Methods and Materials

### Participants

Forty-seven patients with GAD were recruited from the mental health center of Chengdu, Sichuan, China. All patients were determined by consensus of two experienced psychiatrists by using the Structured Clinical Interview for DSM-IV (patient edition) (32). Clinical states of the patients were evaluated using the Hamilton anxiety scale (HAMA). Data from one patient was deleted because the BSV is extremely lower than the others (< mean-5 SD). The exclusion criteria included schizophrenia, mental retardation, or personality disorder, history of loss of consciousness, substance abuse, and serious medical or neurological illness. The HC group was composed of 38 age-, gender-, education-, mean frame-wise displacement (FD) (33)-matched healthy participants. The Structured Clinical Interview for DSM-IV non-patient version was employed to ensure the absence of psychiatric illnesses in the HCs. The HCs did not finish the HAMA scale test because the test was only obtained in hospital by two well-trained psychiatrists. None of the HCs had a history of serious medical or neuropsychiatric illness or a family history of major psychiatric or neurological illness in their first-degree relatives. All participants (including GADs and HCs) finished the Chinese vision of Trait Anxiety Inventory (TAI) questionnaire (34), which is often used in clinical application in the diagnostic work-up of mental disorders and has good validity and reliability (35, 36).

Ultimately, 47 patients with GAD and 38 HC were included in the study (Table 1). Written informed consent, approved by the research ethical committee of the School of Life Science and Technology at University of Electronic Science and Technology of China, was obtained from each participant.

**Table 1:** Demographic information and characteristics of patients with GAD and HCs.

**Variables (Mean ± SD)**	**GAD**	**HC**	***P*-value**
Gender (Male/Female)	47 (17/30)	38 (19/19)	0.200a
Age (years)	38.38 ± 9.08	35.24 ± 10.34	0.139b
Education (years)	11.30 ± 3.64	12.37 ± 3.89	0.195b
mean FD (mm)	0.0923 ± 0.0470	0.1049 ± 0.0555	0.261b
Course of illness (months)	61.96 ± 73.98	-	-
HAMA score	24.28 ± 6.583	-	-
TAI	55.04 ± 8.698	41.28 ± 5.43	<0.0001 b
Medication load index	1.40 ± 0.85	-	-
**ANTIANXIETY MEDICATIONS, NO. OF PATIENTS**			
Fluoxetine	1	-	-
Sertraline	5	-	-
Paroxetine	13	-	-
Citalopram	1	-	-
Escitalopram	9	-	-
Fluvoxamine	1	-	-
Venlafaxine	5	-	-
Duloxetine	1	-	-
Mirtazapine	8	-	-

*SD, standard deviation; GAD, generalized anxiety disorder; HCs, healthy controls; HAMA, Hamilton Anxiety Rating Scale; TAI, Spielberger Trait Anxiety Inventory. a Chi-square test; b Independent-sample t-test*.

### Data Acquisition

MRI data were acquired using a 3.0T GE 750 scanner (General Electric, Fairfield, Connecticut, USA) equipped with high-speed gradients. An 8-channel prototype quadrature birdcage head coil fitted with foam padding was applied to minimize the head motion. Ear plugs were used to minimize the scanner noise. Participants were instructed to simply rest with their eyes closed, minds relaxed, awake, and motionless. Functional images were acquired using a gradient-recalled echo-planar imaging (EPI) sequence. The parameters were as follows: repetition time/echo time = 2,000 ms/30 ms, 90° flip angle, bandwidth = 250 Hz/pixel, 43 axial slices (3.2 mm slice thickness without gap), 64 × 64 matrix, and 22 cm field of view. For each participant, 255 volumes were obtained.

### Data Preprocessing

Functional images were preprocessed using the Data Processing Assistant for resting-state fMRI (DPARSF 2.2, http://www.restfmri.net/forum/DPARSF). The first five volumes were discarded to ensure signal equilibrium and for the participants to familiarize themselves with the scanning environment. The remaining 250 images were slice-time corrected, spatially aligned, and spatially normalized to the Montreal Neurological Institute (MNI) EPI template and resampled to 3 × 3 × 3 mm^3^ voxels. After signal detrending, the images were spatially smoothed (8 mm full width at half maximum Gaussian kernel). Afterwards, Friston 24 head motion parameters, white matter signal, and cerebrospinal fluid signal were further regressed out. Finally, signal was filtered at 0.04–0.07 Hz because of the less noise contamination within this frequency range (37). The frame-wise displacement (FD) was used to represent instantaneous head motion. The mean FD of each participant was <0.5 mm.

### Brain Signal Variability

Firstly, before calculating the BSV, we performed the temporal normalization for each voxel during the entire time of 500 s. The purpose of this step is to eliminate the contamination of the mean signal (14). Secondly, the SD which is simply the square root transformation of variance, was calculated in each voxel cross time series by using a custom-built function in MATLAB (The MathWorks, Inc.). According to Garrett's study, the SD of BOLD signal is temporal variability called BSV (14). The Anatomical Automatic Labeling 90 template was transformed to a binary mask and used to constrain the calculation in the gray matter (21).

### Statistical Analysis

Two-sample *t*-test was used to assess the difference of BSV between the GAD and HC groups. Multiple-comparison correction was performed on the contrast brain map via the false discovery rate approach (*p* < 0.05). Pearson correlations were calculated between the BSV and TAI score in the two groups, correspondingly, with age, sex, education, and mean FD as control variables. The correlation analysis was performed under the regions with significantly different BSVs between the two groups. We adopted the cross-voxel correlation (21, 38) to evaluate the spatial correlation between the correlation maps of the GAD and HC groups. As suggested by Liang et al., the two 3D maps of correlation coefficient were first transformed into columns and then transformed into z-score by minus mean then divided by SD. Pearson correlation was finally computed between these two columns of data. Then, we built a linear mixed-effects model with State-Trait Anxiety Inventory (STAI) score as a factor to show the differences in these correlations between BSV and STAI score in the two groups.

## Results

### Decreased BSV in Patients With GAD

**Figure 1:**
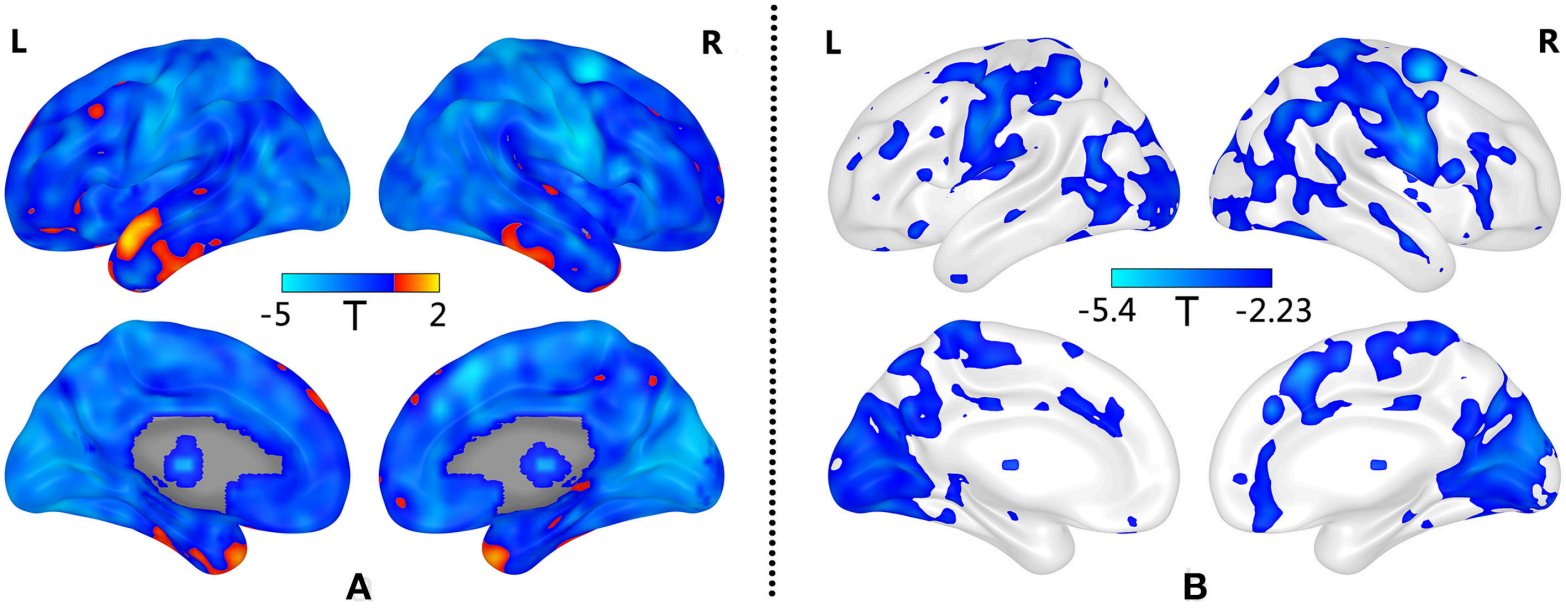
Decreased BSV in widespread brain regions of GAD patients. **(A)** Decreased BSV across the brain in GAD; **(B)** Significantly decreased BSV in patients with GAD is primarily located in the visual network, sensorimotor network, frontoparietal network, limbic system and thalamus (FDR correction, *p* < 0.05). L: left; R: right.

Compared with the HC group, patients with GAD show decreased BSV across the widespread brain regions (Figure 1A). Significantly low BSV is primarily located in the visual network, sensorimotor network, frontoparietal network, limbic system, and thalamus (Figure 1B and Table 2).

**Table 2:** Decreased BSV in patients with GAD compared with HC group.

	**Brain regions**	**BA**	**Cluster size**	**T value**	**MNI coordinates**		
					**X**	**Y**	**Z**
Cluster 1	Right superior frontal gyrus	5\6\17\18\32	13576	−5.4	21	0	57
	Bilateral postcentral gyrus						
	Bilateral supplementary motor area						
	Bilateral precentral gyrus						
	Bilateral occipital cortex						
	Dorsal anterior cingulate cortex						
Cluster 2	Right medial frontal gyrus	10	57	−4.10	9	66	27
Cluster 3	Left thalamus		194	−3.65	−3	−9	9
	Right caudate						
Cluster 4	Right inferior temporal gyrus	20\21	83	−3.61	60	−6	21
	Right middle temporal gyrus						
Cluster 5	Medial frontal gyrus	24	251	−3.45	6	39	−18
	Bilateral anterior cingulate						
Cluster 6	Left middle frontal gyrus	46	144	−3.42	−33	39	15

*BA, Brodmann's area; MNI, Montreal Neurological Institute; X, Y, Z, coordinates of primary peak locations in the MNI space; T statistical value of peak voxel showing BSV decreased. All the clusters survived p < 0.05, FDR corrected, and a minimum cluster size of 50 voxels. Degree of freedom = 78*.

### Correlations Between BSV and Anxiety Severity in GAD and HC

**Figure 2:**
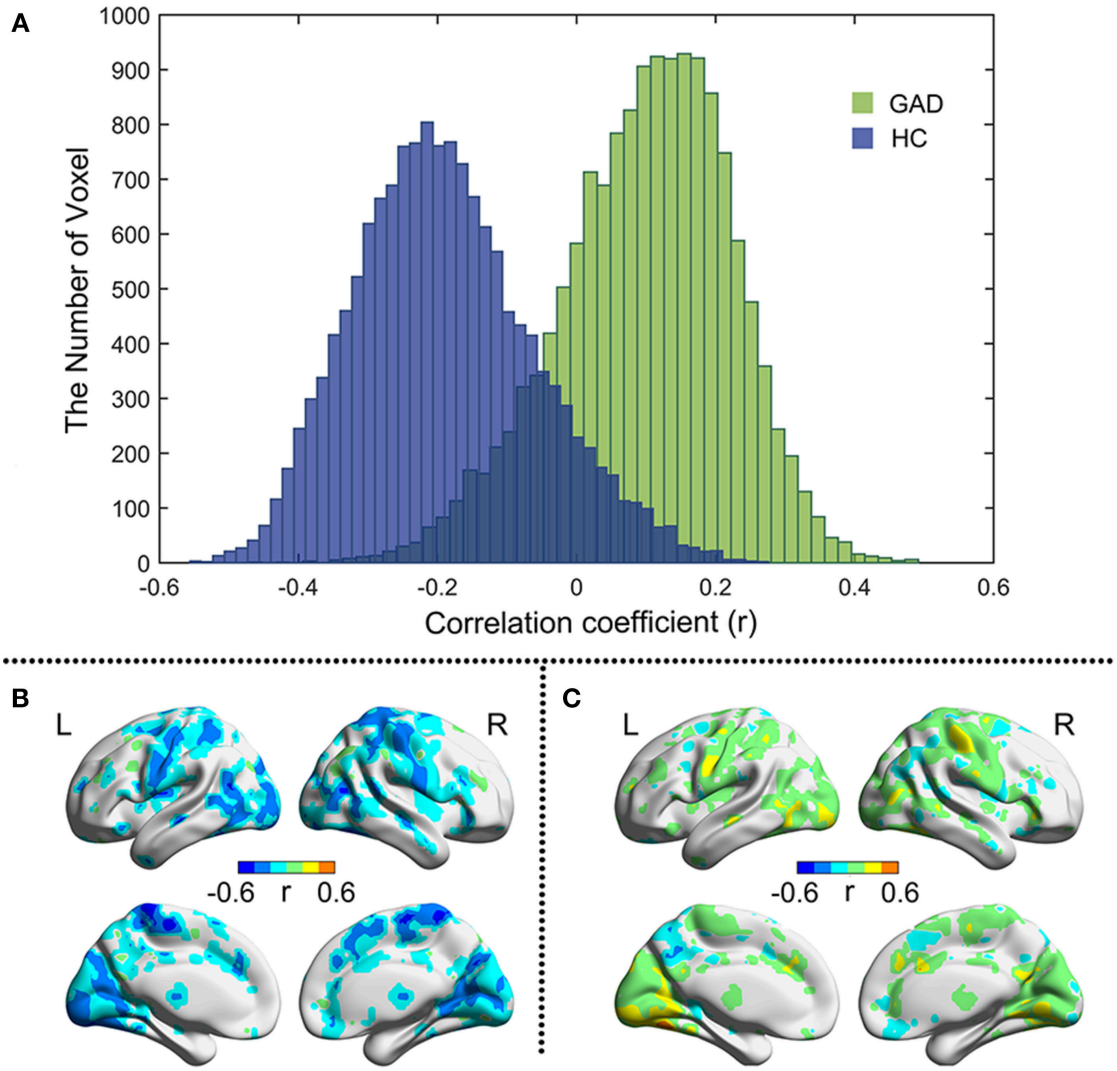
Pearson correlation between BSV and TAI score in two groups. **(A)** Histogram illustrates voxel-wise correlations for GAD (green) and HC (blue) groups, respectively; **(B)** The r map of HC group; **(C)** The r map of GAD group. L: left; R: right.

**Figure 3:**
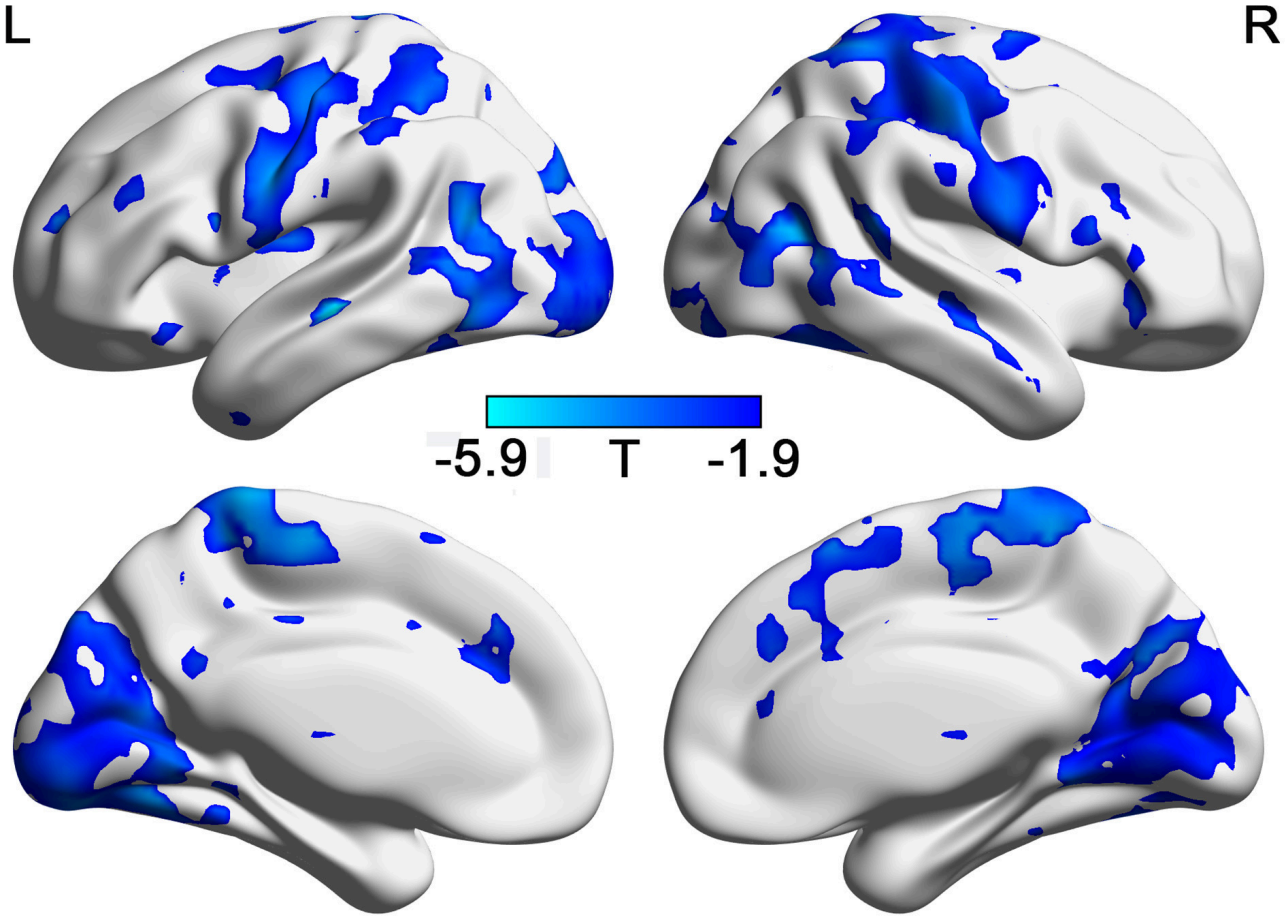
The different relationship with STAI in GADs and in HCs. (FDR correction, *p* < 0.05). L: left; R: right.

As shown in Figure 2A, the correlation between BSV and TAI score in the HC group is mainly negative, whereas that in the GAD group is mainly positive. Figures 2B,C show the correlation map for the HC and GAD groups, respectively. The correlation coefficients in the HC group and GAD group are negatively related to each other (*r* = −0.285, *p* = 4.12e−274). In other words, if the correlation between BSV and TAI score in the HC group is negative in one voxel, that correlation in the GAD group is positive in that voxel. The differences of correlations between BSV and STAI score in the two groups are also mainly located in the visual network, sensorimotor network, and frontoparietal network (Figure 3).

## Discussion

To the best of our knowledge, this work is the first study on the abnormal BSV in patients with GAD. In accordance with other studies in mental disorders, we observed widely decreased BSV in patients with GAD compared with HCs, suggesting multiple deficits in neural systems of GAD. Interestingly, we observed opposite distributions of linear relationship between TAI score and BSV in the GAD and HC groups, indicating a non-linear relationship between anxiety level and BSV. In other words, the chaotic brain might be not always the healthy one.

### Decreased Temporal Variability in Patients With GAD

In line with reduced BSV in other mental disorders, the current study revealed widely decreased BSV in patients with GAD, arguing that reduced BSV is a general characteristic of mental disorders. Recent studies have shown that low amplitude of low-frequency fluctuation (ALFF) in the left supplementary motor area, right middle occipital gyrus, cerebellum, prefrontal-limbic system, and thalamus is associated with high-trait anxiety (32, 39, 40). Because ALFF is the square root of power (41) and power is equivalent to BSV (21). The BSV may reflect similar neural mechanisms to ALFF but in a non-linear way (21). Specifically, decreased BSV is associated with small potential kinetic energy to handle external environmental demands (16, 22) and low ability to transfer between different brain states (i.e., rest state and task state) (42). In patients with anxiety disorder, the functional deficit of environmental detection and the inflexible functional brain configurations has been found (31, 43). Therefore, the widely decreased BSV in patients with GAD may be associated with limited ability to adapt different external conditions and inflexible pattern of information transfer.

Low BSV in GAD is mainly located in the visual cortex, somatosensory cortex, anterior cingulate cortex (ACC), and thalamus. Multiple studies have documented deficits in the visual cortex in different anxiety disorders (44–46). Visual cortex plays a core role in the social brain network during visual and emotion processing (47). Visual cortex is also involved in the processing of fear generalization (48), which may greatly contribute to the GAD psychopathology (49, 50). The postcentral gyrus, as a critical substrate of interoceptive processing (51), is responsible for receiving, integrating, and interpreting most of the sensory information transmitted by the thalamus (52, 53). As shown by many studies, the thalamus is also strongly involved in interoceptive awareness (54–57). Abnormal connectivity between the postcentral gyrus and thalamus has been found in panic disorder, which may be associated with the typical symptoms (e.g., the extreme feeling of heartbeat) of panic disorder (6). In general, as a hub of the interoceptive network, the ACC participates in mediating visceromotor activity and has projections into motor systems (56). Decreased functional connections between the ACC and thalamus was negatively correlated with HAMA in GAD, which may cause some somatic disturbed symptoms, such as rapid heart rate, low skin conductance, and difficulty breathing (58). Above all, the reduction of variability in these brain areas may be related to biased perception in processing exogenous and endogenous information (6, 59), which is implicated in the GAD pathological mechanism.

Reduced BSV in patients with GAD is also found in the dorsolateral prefrontal cortex and inferior parietal lobe, which are the key brain regions of the frontoparietal network (FPN). The deficiencies in FPN have been found in individuals with high-trait anxiety who have poor cognitive control and are easily distracted by emotional distractors from external environment (60, 61). Individuals with high anxiety generally require additional attentional control even in the absence of threat-related stimuli (62). The FPN is also a flexible hub during brain state transition (63). Low variability in the FPN may be associated with low ability to transfer between different brain states (42). Therefore, low BSV in the FPN may be associated with inflexible information to transform patterns facing the external world in patients with GAD.

### Opposite Relationships Between BSV and Anxiety in the GAD and HC Groups

The opposite correlations between the BSV and TAI score in the HC group and in the GAD group has been found in the present study, indicating that the moderate anxious brain has small BSV. Moderate anxiety is associated with the best performance (64–66), and BSV has been demonstrated to be closely related to behavior performance (19, 24). In certain brain regions, maintaining great signal stability is also a critical marker of good task performance (22). In other words, the direction of correlation between BSV and behavioral performance depends on both cognition and brain region (21, 24, 67). Therefore, the opposite relationships between BSV and anxiety level may indicate that (1) moderate anxiety is associated with the most stable brain state and the best performance, and (2) increased BSV in subjects with high anxiety and low anxiety may be related to distinctive brain states, leading to different performances. Considering that (1) the dopaminergic system is closely associated with both anxiety and BSV (68, 69), (2) the non-linear relationship between dopaminergic system and behavioral performance (70), and (3) the quadratic relationship between anxiety and behavioral performance (66), the opposite BSV-anxiety relationship may reflect different neural dynamical configurations mediated by the dopaminergic system in patients with GAD and healthy people. Furthermore, the negative correlation between the HC group and GAD group suggests that the neuropsychological association in HC and patients with GAD may be driven by the same mechanism rather than independent from each other. This hypothesis deserves further investigations.

Although increased anxious levels in GADs showed increased BSV which indicated the great capacity of detection, the mechanism of this seemingly increasing ability can be totally different from healthy participants. In patients with GAD, the excessive activation is an apparent clinical symptom (1, 2), which might be associated with the high detection. In addition, this conclusion still needs further research. Meanwhile, the great BSV in some regions can be a compensation mechanism of an inefficient brain (14).

Overall, the opposite correlations between the BSV and TAI score in two groups indicate close relationships among dopaminergic system, behavioral performance, anxiety level, and BSV. These relationships can be described by the U law. Therefore, BSV is not always linearly related to brain health (24). These results provide valuable insights into understanding the relationship between BSV and health.

## Limitations

Some limitations remain in this study. First, the sample is relatively small (*n* = 85), impeding the uncovering of a strong correlation between BSV and anxiety level. Second, the HAMA score is lacking in the HC group because these participants were tested at the university rather than at the hospital, hindering the full investigation of clinical relevance of BSV. Third, some undiagnosed comorbid psychological disorders may exist in this sample, which may mix the true relationship between BSV and anxiety. A large sample with accurate diagnosis and complete scale collection is needed in future studies.

## Conclusion

In summary, the decreased BSV in patients with GAD and different neuropsychological relationships in patients with GAD and HCs may reveal a novel neurodynamic mechanism, suggesting that the chaotic brain is not always the healthy one.

## Data Availability

The datasets generated for this study are available on request to the corresponding author.

## Ethics Statement

All procedures followed were in accordance with the ethical standards of the responsible committee on human experimentation (institutional and national) and with the Helsinki Declaration of 1975 and the applicable revisions at the time of investigation. Informed consent was obtained from all patients included in the study.

## Author Contributions

LL, YW, and HC designed the study. LY, WC, DL, and HX wrote the computing scripts. QC and ZH interviewed all patients by using the DSM-IV. LL managed the literature searches and analyses, acquired, and analyzed the data, and wrote the first manuscript draft. All authors contributed and have approved the final manuscript. The authors thank all subjects participating in this study.

### Conflict of Interest Statement

The authors declare that the research was conducted in the absence of any commercial or financial relationships that could be construed as a potential conflict of interest.

## References

[1] Shin LM Liberzon I. The neurocircuitry of fear, stress and anxiety disorders. Neuropsychopharmacology. (2010) 35:169–91. 10.1038/npp.2009.83 19625997 PMC3055419

[2] American Psychiatric Association Diagnostic and Statistical Manual of Mental Disorders. 5th ed. Washington, DC: American Psychiatric Association (2013). p. 280.

[3] Makovac E Meeten F Watson DR Herman A Garfinkel SN D. Alterations in amygdala-prefrontal functional connectivity account for excessive worry and autonomic dysregulation in generalized anxiety disorder. Biol Psychiatry. (2016) 80:786–95. 10.1016/j.biopsych.2015.10.013 26682467

[4] Cui Q Vanman EJ Long Z Pang Y Chen Y Wang Y. Social anxiety disorder exhibit impaired networks involved in self and theory of mind processing. Soc Cogn Affect Neurosci. (2017) 12:1284–95. 10.1093/scan/nsx050 28398578 PMC5597891

[5] Liu F Guo W Fouche J-P Wang Y Wang W Ding J. Multivariate classification of social anxiety disorder using whole brain functional connectivity. Brain Struct Func. (2015) 220:101–15. 10.1007/s00429-013-0641-4 24072164

[6] Cui H Zhang J Liu Y Li Q Li H Zhang L. Differential alterations of resting-state functional connectivity in generalized anxiety disorder and panic disorder. Hum Brain Mapp. (2016) 37:1459–73. 10.1002/hbm.23113 26800659 PMC6867341

[7] Xia L Li S Wang T Guo Y Meng L Feng Y. Spontaneous alterations of regional brain activity in patients with adult generalized anxiety disorder. Neuropsychiatr Dis Treat. (2017) 13:1957–65. 10.2147/NDT.S133853 28790831 PMC5530096

[8] Liu F Wang Y Li M Wang W Li R Zhang Z. Dynamic functional network connectivity in idiopathic generalized epilepsy with generalized tonic-clonic seizure. Hum Brain Mapp. (2016) 38:957–73. 10.1002/hbm.23430 27726245 PMC6866949

[9] Nelson B McGorry PD Wichers M Wigman JTW Hartmann JA. Moving from static to dynamic models of the onset of mental disorder. JAMA Psychiatry. (2017) 74:528. 10.1001/jamapsychiatry.2017.0001 28355471

[10] Barber AD Lindquist MA DeRosse P Karlsgodt KH. Dynamic functional connectivity states reflecting psychotic-like experiences. Biol Psychiatry Cogn Neurosci Neuroimaging. (2018) 3:443–53. 10.1016/j.bpsc.2017.09.008 29735154 PMC5944620

[11] Pool R. Is it healthy to be chaotic? Science. (1989) 243:604–7. 10.1126/science.2916117 2916117

[12] Biswal B Zerrin Yetkin F Haughton VM Hyde JS. Functional connectivity in the motor cortex of resting human brain using echo-planar MRI. Magn Resonance Med. (1995) 34:537–41. 10.1002/mrm.1910340409 8524021

[13] Stein T. Bacillus subtilis antibiotics: structures, syntheses and specific functions. Mol Microbiol. (2005) 56:845–57. 10.1111/j.1365-2958.2005.04587.x 15853875

[14] Garrett DD Kovacevic N McIntosh AR Grady CL. Blood oxygen level-dependent signal variability is more than just noise. J Neurosci. (2010) 30:4914–21. 10.1523/JNEUROSCI.5166-09.2010 20371811 PMC6632804

[15] Wutte MG Smith MT Flanagin VL Wolbers T. Physiological signal variability in hMT+ reflects performance on a direction discrimination task. Front Psychol. (2011) 2:185. 10.3389/fpsyg.2011.00185 21852978 PMC3151615

[16] Garrett DD Samanez-Larkin GR MacDonald SWS Lindenberger U McIntosh AR Grady CL. Moment-to-moment brain signal variability: a next frontier in human brain mapping? Neurosci Biobehav Rev. (2013) 37:610–24. 10.1016/j.neubiorev.2013.02.015 23458776 PMC3732213

[17] He BJ. Scale-free properties of the functional magnetic resonance imaging signal during rest and task. J Neurosci. (2011) 31:13786–95. 10.1523/JNEUROSCI.2111-11.2011 21957241 PMC3197021

[18] Leo A Bernardi G Handjaras G Bonino D Ricciardi E Pietrini P. Increased BOLD variability in the parietal cortex and enhanced parieto-occipital connectivity during tactile perception in congenitally blind individuals. Neural Plast. (2012) 2012:720278. 10.1155/2012/720278 22792493 PMC3388315

[19] Wang Y-F Dai G-S Liu F Long Z-L Yan JH Chen H-F. Steady-state BOLD response to higher-order cognition modulates low-frequency neural oscillations. J Cogn Neurosci. (2015) 27:2406–15. 10.1162/jocn_a_00864 26284992

[20] Wang Y-F Liu F Long Z-L Duan X-J Cui Q Yan JH. Steady-state BOLD response modulates low frequency neural oscillations. Sci Rep. (2015) 4:7376. 10.1038/srep07376 25488025 PMC4260215

[21] Wang Y Chen W Ye L Biswal BB Yang X Zou Q. Multiscale energy reallocation during low-frequency steady-state brain response. Hum Brain Mapp. (2018) 39:2121–32. 10.1002/hbm.23992 29389047 PMC6866265

[22] Garrett DD Kovacevic N McIntosh AR Grady CL. The importance of being variable. J Neurosci. (2011) 31:4496–503. 10.1523/JNEUROSCI.5641-10.2011 21430150 PMC3104038

[23] Garrett DD McIntosh AR Grady CL. Brain signal variability is parametrically modifiable. Cereb Cortex. (2014) 24:2931–40. 10.1093/cercor/bht150 23749875 PMC4193462

[24] Nomi JS Bolt TS Ezie CEC Uddin LQ Heller AS. Moment-to-moment BOLD signal variability reflects regional changes in neural flexibility across the lifespan. J Neurosci. (2017) 37:5539–48. 10.1523/JNEUROSCI.3408-16.2017 28473644 PMC5452342

[25] Huang Z Zhang J Wu J Qin P Wu X Wang Z. Decoupled temporal variability and signal synchronization of spontaneous brain activity in loss of consciousness: an fMRI study in anesthesia. Neuroimage. (2016) 124:693–703. 10.1016/j.neuroimage.2015.08.062 26343319

[26] Zhang J Cheng W Liu Z Zhang K Lei X Yao Y. Neural, electrophysiological and anatomical basis of brain- network variability and its characteristic changes in mental disorders. Brain. (2016) 139:2307–21. 10.1093/brain/aww143 27421791

[27] Takahashi T. Complexity of spontaneous brain activity in mental disorders. Prog Neuro-Psychopharmacol Biol Psychiatry. (2013) 45:258–66. 10.1016/j.pnpbp.2012.05.001 22579532

[28] Protzner AB Valiante T Kovacevic N McCormick C McAndrews MP. Hippocampal signal complexity in mesial temporal lobe epilepsy: a noisy brain is a healthy brain. Arch Ital Biol. (2010) 148:289–97. 21175015

[29] Friedman BH Thayer JF. Autonomic balance revisited: panic anxiety and heart rate variability. J Psychosom Res. (1998) 44:133–51. 10.1016/S0022-3999(97)00202-X 9483470

[30] Ottaviani C Watson DR Meeten F Makovac E Garfinkel SN Critchley HD. Neurobiological substrates of cognitive rigidity and autonomic inflexibility in generalized anxiety disorder. Biol Psychol. (2016) 119:31–41. 10.1016/j.biopsycho.2016.06.009 27345596

[31] Fonzo GA Etkin A. Affective neuroimaging in generalized anxiety disorder: an integrated review. Dialogues Clin Neurosci. (2017) 19:169–79. 28867941 10.31887/DCNS.2017.19.2/gfonzo PMC5573561

[32] Yuan C Zhu H Ren Z Yuan M Gao M Zhang Y. Precuneus-related regional and network functional deficits in social anxiety disorder: a resting-state functional MRI study. Compr Psychiatry. (2018) 82:22–9. 10.1016/j.comppsych.2017.12.002 29367059

[33] Power JD Barnes KA Snyder AZ Schlaggar BL Petersen SE. Spurious but systematic correlations in functional connectivity MRI networks arise from subject motion. Neuroimage. (2012) 59:2142–54. 10.1016/j.neuroimage.2011.10.018 22019881 PMC3254728

[34] Speilberger CD Gorsuch RL Lushene R Vagg PR Manual for the State-Trait Anxiety Inventory (Form Y1 - Y2). Palo Alto, CA: Consulting Psychologists (1983)

[35] Shek DTL. The Chinese version of the state-trait anxiety inventory: its relationship to different measures of psychological well-being. J Clin Psychol. (1993) 49:349–58. 8315037 10.1002/1097-4679(199305)49:3<349::aid-jclp2270490308>3.0.co;2-j

[36] Kvaal K Ulstein I Nordhus IH Engedal K. The Spielberger state-trait anxiety inventory (STAI): the state scale in detecting mental disorders in geriatric patients. Int J Geriatr Psychiatry. (2005) 20:629–34. 10.1002/gps.1330 16021666

[37] Glerean E Salmi J Lahnakoski JM Jääskeläinen IP Sams M. Functional magnetic resonance imaging phase synchronization as a measure of dynamic functional connectivity. Brain Connect. (2012) 2:91–101. 10.1089/brain.2011.0068 22559794 PMC3624768

[38] Liang X Zou Q He Y Yang Y. Coupling of functional connectivity and regional cerebral blood flow reveals a physiological basis for network hubs of the human brain. Proc Nat Acad Sci. (2013) 110:1929–34. 10.1073/pnas.1214900110 23319644 PMC3562840

[39] Wang W Hou J Qian S Liu K Li B Li M. Aberrant regional neural fluctuations and functional connectivity in generalized anxiety disorder revealed by resting-state functional magnetic resonance imaging. Neurosci Lett. (2016) 624:78–84. 10.1016/j.neulet.2016.05.005 27163197

[40] Yin P Zhang M Hou X Tan Y Fu Y Qiu J. The brain structure and spontaneous activity baseline of the behavioral bias in trait anxiety. Behav Brain Res. (2016) 312:355–61. 10.1016/j.bbr.2016.06.036 27340090

[41] Yang H Long X-Y Yang Y Yan H Zhu C-Z Zhou X-P. Amplitude of low frequency fluctuation within visual areas revealed by resting-state functional MRI. Neuroimage. (2007) 36:144–52. 10.1016/j.neuroimage.2007.01.054 17434757

[42] Garrett DD Kovacevic N McIntosh AR Grady CL. The modulation of BOLD variability between cognitive states varies by age and processing speed. Cerebral Cortex. (2013) 23:684–93. 10.1093/cercor/bhs055 22419679 PMC3823571

[43] de Rooij SR Schene AH Phillips DI Roseboom TJ. Depression and anxiety: associations with biological and perceived stress reactivity to a psychological stress protocol in a middle-aged population. Psychoneuroendocrinology. (2010) 35:866–77. 10.1016/j.psyneuen.2009.11.011 20031333

[44] Etkin A Wager TD. Functional neuroimaging of anxiety: a meta-analysis of emotional processing in PTSD, social anxiety disorder, and specific phobia. Am J Psychiatry. (2007) 164:1476–88. 10.1176/appi.ajp.2007.07030504 17898336 PMC3318959

[45] Gentili C Gobbini MI Ricciardi E Vanello N Pietrini P Haxby JV. Differential modulation of neural activity throughout the distributed neural system for face perception in patients with Social Phobia and healthy subjects. Brain Res Bull. (2008) 77:286–92. 10.1016/j.brainresbull.2008.08.003 18771714

[46] Syal S Hattingh CJ Fouché J-P Spottiswoode B Carey PD Lochner C. Grey matter abnormalities in social anxiety disorder: a pilot study. Metab Brain Dis. (2012) 27:299–309. 10.1007/s11011-012-9299-5 22527992

[47] Sabatinelli D Bradley MM Fitzsimmons JR Lang PJ. Parallel amygdala and inferotemporal activation reflect emotional intensity and fear relevance. Neuroimage. (2005) 24:1265–70. 10.1016/j.neuroimage.2004.12.015 15670706

[48] Lissek S Bradford DE Alvarez RP Burton P Espensen-Sturges T Reynolds RC. Neural substrates of classically conditioned fear-generalization in humans: a parametric fMRI study. Soc Cogn Affect Neurosci. (2014) 9:1134–42. 10.1093/scan/nst096 23748500 PMC4127021

[49] Lissek S. Toward an account of clinical anxiety predicated on basic, neurally-mapped mechanisms of pavlovian fear-learning: the case for conditioned overgeneralization. Depress Anxiety. (2012) 29:257–63. 10.1002/da.21922 22447565 PMC4194209

[50] Lissek S Kaczkurkin AN Rabin S Geraci M Pine DS Grillon C. Generalized anxiety disorder is associated with overgeneralization of classically conditioned-fear. Biol Psychiatry. (2014) 75:909–15. 10.1016/j.biopsych.2013.07.025 24001473 PMC3938992

[51] Critchley HD Wiens S Rotshtein P Öhman A Dolan RJ. Neural systems supporting interoceptive awareness. Nat Neurosci. (2004) 7:189–95. 10.1038/nn1176 14730305

[52] Nelson AJ Chen R. Digit somatotopy within cortical areas of the postcentral gyrus in humans. Cereb Cortex. (2008) 18:2341–51. 10.1093/cercor/bhm257 18245039

[53] Northoff G Unlocking the Brain, Volume 2: Consciousness. New York, NY: Oxford University Press (2013).

[54] Rieck RW Ansari MS Whetsell WO Deutch AY Kessler RM. Distribution of dopamine d2-like receptors in the human thalamus: autoradiographic and PET studies. Neuropsychopharmacology. (2004) 29:362–72. 10.1038/sj.npp.1300336 14627996

[55] Pollatos O Schandry R Auer DP Kaufmann C. Brain structures mediating cardiovascular arousal and interoceptive awareness. Brain Res. (2007) 1141:178–87. 10.1016/j.brainres.2007.01.026 17296169

[56] Khalsa SS Rudrauf D Feinstein JS Tranel D. The pathways of interoceptive awareness. Nat Neurosci. (2009) 12:1494–6. 10.1038/nn.2411 19881506 PMC2787640

[57] Cho YT Fromm S Guyer AE Detloff A Pine DS Fudge JL. Nucleus accumbens, thalamus and insula connectivity during incentive anticipation in typical adults and adolescents. Neuroimage. (2013) 66:508–21. 10.1016/j.neuroimage.2012.10.013 23069809 PMC3949208

[58] Hoehn-Saric R McLeod DR Funderburk F Kowalski P. Somatic symptoms and physiologic responses in generalized anxiety disorderand panic disorder. Arch Gen Psychiatry. (2004) 61:913. 10.1001/archpsyc.61.9.913 15351770

[59] Wang Y Zhu L Zou Q Cui Q Liao W Duan X. Frequency dependent hub role of the dorsal and ventral right anterior insula. Neuroimage. (2018) 165:112–7. 10.1016/j.neuroimage.2017.10.004 28986206

[60] Pacheco-Unguetti AP Acosta A Callejas A Lupiáñez J. Attention and anxiety: different attentional functioning under state and trait anxiety. Psychol Sci. (2010) 21:298–304. 10.1177/0956797609359624 20424060

[61] Hofmann SG Ellard KK Siegle GJ. Neurobiological correlates of cognitions in fear and anxiety: a cognitive-neurobiological information-processing model. Cognit Emot. (2012) 26:282–99. 10.1080/02699931.2011.579414 21806384 PMC3229257

[62] Basten U Stelzel C Fiebach CJ. Trait anxiety modulates the neural efficiency of inhibitory control. J Cogn Neurosci. (2011) 23:3132–45. 10.1162/jocn_a_00003 21391763

[63] Cole MW Reynolds JR Power JD Repovs G Anticevic A Braver TS. Multi-task connectivity reveals flexible hubs for adaptive task control. Nat Neurosci. (2013) 16:1348–55. 10.1038/nn.3470 23892552 PMC3758404

[64] Sonstroem RJ Bernardo P Intraindividual pregame state anxiety and basketball performance: a re-examination of the inverted-U curve. J Sport Psychol. (1982) 4:235–45. 10.1123/jsp.4.3.235

[65] Gould D Petlichkoff L Simons J Vevera M Relationship between competitive state anxiety—inventory-2 subscale scores and pistol shooting performance. J Sport Psychol. (1987) 9:33–42. 10.1123/jsp.9.1.33

[66] Raglin JS Turner PE Anxiety and performance in track and field athletes: a comparison of the inverted-U hypothesis with zone of optimal function theory. Pers Individ Dif. (1993) 14:163–71. 10.1016/0191-8869(93)90186-7

[67] Armbruster-Genc DJN Ueltzhoffer K Fiebach CJ. Brain signal variability differentially affects cognitive flexibility and cognitive stability. J Neurosci. (2016) 36:3978–87. 10.1523/JNEUROSCI.2517-14.2016 27053205 PMC6705511

[68] Guitart-Masip M Salami A Garrett D Rieckmann A Lindenberger U Bäckman L. BOLD variability is related to dopaminergic neurotransmission and cognitive aging. Cereb Cortex. (2016) 26:2074–83. 10.1093/cercor/bhv029 25750252

[69] Kim S Shou J Abera S Ziff EB. Sucrose withdrawal induces depression and anxiety-like behavior by Kir2. 1 upregulation in the nucleus accumbens. Neuropharmacology. (2018) 130:10–7. 10.1016/j.neuropharm.2017.11.041 29191750

[70] Cools R D'Esposito M. Inverted-U shaped dopamine actions on human working memory and cognitive control. Biol. Psychiatry. (2011) 69:e113–25. 10.1016/j.biopsych.2011.03.028 21531388 PMC3111448

